# Prevalence of SARS-CoV-2 and Influenza Coinfection and Clinical Characteristics Among Children and Adolescents Aged <18 Years Who Were Hospitalized or Died with Influenza — United States, 2021–22 Influenza Season

**DOI:** 10.15585/mmwr.mm7150a4

**Published:** 2022-12-16

**Authors:** Katherine Adams, Katie J. Tastad, Stacy Huang, Dawud Ujamaa, Krista Kniss, Charisse Cummings, Arthur Reingold, Jeremy Roland, Elizabeth Austin, Breanna Kawasaki, James Meek, Kimberly Yousey-Hindes, Evan J. Anderson, Kyle P. Openo, Libby Reeg, Lauren Leegwater, Melissa McMahon, Erica Bye, Mayvilynne Poblete, Zachary Landis, Nancy L. Spina, Kerianne Engesser, Nancy M. Bennett, Maria A. Gaitan, Eli Shiltz, Nancy Moran, Melissa Sutton, Nasreen Abdullah, William Schaffner, H. Keipp Talbot, Kristen Olsen, Holly Staten, Christopher A. Taylor, Fiona P. Havers, Carrie Reed, Alicia Budd, Shikha Garg, Alissa O’Halloran, Lynnette Brammer

**Affiliations:** ^1^Influenza Division, National Center for Immunization and Respiratory Diseases, CDC; ^2^Goldbelt Professional Services, Herndon, Virginia; ^3^General Dynamics Information Technology, Atlanta, Georgia; ^4^University of California, Berkeley, California Emerging Infections Program, Oakland, California; ^5^Colorado Department of Public Health and Environment; ^6^Connecticut Emerging Infections Program, Yale School of Public Health, New Haven, Connecticut; ^7^Departments of Pediatrics and Medicine, Emory University School of Medicine, Atlanta, Georgia; ^8^Georgia Emerging Infections Program, Georgia Department of Public Health, Atlanta, Georgia; ^9^Atlanta Veterans Affairs Medical Center, Atlanta, Georgia; ^10^Division of Infectious Diseases, School of Medicine, Emory University, Atlanta, Georgia; ^11^Michigan Department of Health and Human Services; ^12^Minnesota Department of Health; ^13^New Mexico Emerging Infections Program, University of New Mexico, Albuquerque, New Mexico; ^14^New York State Department of Health; ^15^University of Rochester School of Medicine and Dentistry, Rochester, New York; ^16^Ohio Department of Health; ^17^Oregon Health Authority; ^18^Department of Health Policy, Vanderbilt University Medical Center, Nashville, Tennessee; ^19^Salt Lake County Health Department, Salt Lake City, Utah; ^20^Division of Viral Diseases, National Center for Immunization and Respiratory Diseases, CDC.

The 2022–23 influenza season shows an early rise in pediatric influenza-associated hospitalizations (*1*). SARS-CoV-2 viruses also continue to circulate (*2*). The current influenza season is the first with substantial co-circulation of influenza viruses and SARS-CoV-2 (*3*). Although both seasonal influenza viruses and SARS-CoV-2 can contribute to substantial pediatric morbidity (*3*–*5*), whether coinfection increases disease severity compared with that associated with infection with one virus alone is unknown. This report describes characteristics and prevalence of laboratory-confirmed influenza virus and SARS-CoV-2 coinfections among patients aged <18 years who had been hospitalized or died with influenza as reported to three CDC surveillance platforms during the 2021–22 influenza season. Data from two Respiratory Virus Hospitalizations Surveillance Network (RESP-NET) platforms (October 1, 2021–April 30, 2022),^§^ and notifiable pediatric deaths associated^¶^ with influenza virus and SARS-CoV-2 coinfection (October 3, 2021–October 1, 2022)** were analyzed. SARS-CoV-2 coinfections occurred in 6% (32 of 575) of pediatric influenza-associated hospitalizations and in 16% (seven of 44) of pediatric influenza-associated deaths. Compared with patients without coinfection, a higher proportion of those hospitalized with coinfection received invasive mechanical ventilation (4% versus 13%; p = 0.03) and bilevel positive airway pressure or continuous positive airway pressure (BiPAP/CPAP) (6% versus 16%; p = 0.05). Among seven coinfected patients who died, none had completed influenza vaccination, and only one received influenza antivirals.^††^ To help prevent severe outcomes, clinicians should follow recommended respiratory virus testing algorithms to guide treatment decisions and consider early antiviral treatment initiation for pediatric patients with suspected or confirmed influenza, including those with SARS-CoV-2 coinfection who are hospitalized or at increased risk for severe illness. The public and parents should adopt prevention strategies including considering wearing well-fitted, high-quality masks when respiratory virus circulation is high and staying up-to-date with recommended influenza and COVID-19 vaccinations for persons aged ≥6 months.

CDC collects data on influenza-associated hospitalizations through the Influenza Hospitalization Surveillance Network (FluSurv-NET), a population-based RESP-NET system that includes more than 250 acute care hospitals.^§§^ Since March 2020, CDC has also collected data on COVID-19–associated hospitalizations through another RESP-NET platform, the COVID-19–associated Hospitalization Surveillance Network (COVID-NET). Influenza and SARS-CoV-2 testing^¶¶^ is driven by clinician decisions or hospital testing policies, with laboratory, clinical, and notifiable disease database sources used to identify patients.*** A FluSurv-NET patient was defined as a person who 1) was a resident of the surveillance catchment area, 2) had a hospital admission during October 1, 2021–April 30, 2022, and 3) had a positive influenza test result within 14 days before or anytime during hospitalization. Coinfected patients were those who met the FluSurv-NET definition and who also 1) had laboratory-confirmed influenza and SARS-CoV-2 infections during the same hospitalization, or 2) were identified through COVID-NET and had a COVID-19–associated hospital admission occurring within 14 days before or after an influenza-associated hospitalization. A patient was considered to have received the current seasonal influenza vaccine if ≥1 dose was administered ≥14 days before the positive influenza test result.^†††^

Data on influenza-associated pediatric deaths that occurred during October 3, 2021–October 1, 2022, were obtained from the Influenza-Associated Pediatric Mortality Surveillance System. A notifiable death is defined as a death in a person aged <18 years resulting from a clinically compatible illness confirmed to be influenza by laboratory testing^§§§^ without a period of complete recovery between illness onset and death. State and local health departments report investigations of these deaths to CDC using a standardized case report form, which includes data on demographic characteristics, influenza testing, bacterial and viral co-detections, clinical diagnoses and complications, medication use, and influenza vaccination. Coinfections with SARS-CoV-2 were identified using the “viral coinfection” field, with either COVID-19 or SARS-CoV-2 indicated in free text.

Across all data sources, patients were eligible to be included in this analysis if they were aged <18 years and had evidence of influenza virus infection. Information on COVID-19 vaccination and antiviral treatment was not included because of lack of systematic ascertainment for patients across data sources. Demographic and clinical characteristics, in-hospital interventions, and outcomes are reported by illness status (influenza and SARS-CoV-2 coinfection and influenza infection alone) as frequencies and proportions, with between-group comparisons analyzed using Pearson’s chi-square tests for hospitalizations and Fisher’s exact tests for deaths. Medians and IQRs are presented for continuous variables, with between-group comparisons analyzed using a Wilcoxon rank sum test. Data were analyzed using SAS software (version 9.4, SAS Institute). These activities were reviewed by CDC and were consistent with applicable federal law and CDC policy.^¶¶¶^

**Hospitalizations.** During October 1, 2021–April 30, 2022, FluSurv-NET identified 575 pediatric influenza-associated hospitalizations, including 32 (6%) patients who were coinfected with SARS-CoV-2 and 543 (94%) who had influenza alone (hereafter, influenza) (Figure). Underlying medical conditions were reported for the majority of hospitalized patients with coinfection (56%) and with influenza (58%) (p = 0.81), whereas receipt of seasonal influenza vaccination was less prevalent among those with coinfections (17%) than among those with influenza (42%) (p = 0.02) (Table 1). A higher proportion of patients with coinfection than with influenza received invasive mechanical ventilation (13% versus 4%; p = 0.03) and BiPAP or CPAP (16% versus 6%; p = 0.05). No significant differences were found between patients with coinfection and with influenza in the prevalence of intensive care unit (ICU) admission (p = 0.20). No in-hospital deaths were identified with FluSurv-NET in either group.

**FIGURE:**
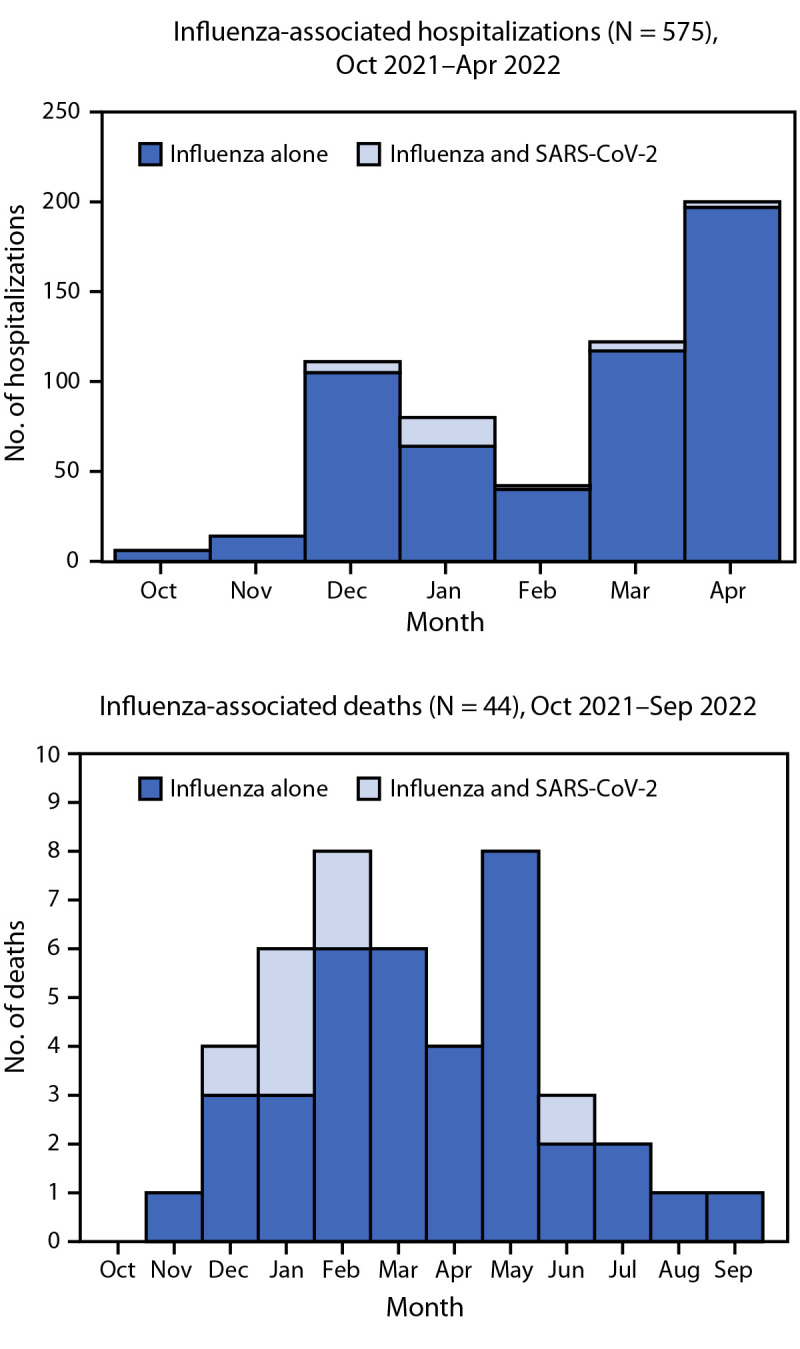
Number of children and adolescents aged <18 years who were hospitalized* or died^†^ with influenza alone and influenza and SARS-CoV-2 coinfections, by month — United States, 2021–22 influenza season * Influenza Hospitalization Surveillance Network; data beyond April 30, 2022, did not include variables required to determine influenza and SARS-CoV-2 coinfection status. ^†^ Influenza-Associated Pediatric Mortality Surveillance System.

**TABLE 1:** Characteristics of children and adolescents aged <18 years hospitalized with laboratory-confirmed influenza and influenza and SARS-CoV-2 coinfections (N = 575) — Influenza Hospitalization Surveillance Network, United States, October 1, 2021–April 30, 2022*

Characteristic	No. of patients (%) with influenza and SARS-CoV-2 coinfection (n = 32)	No. of patients (%) with only influenza (n = 543)	p-value^†^
**Age, yrs, median (IQR)**	3 (1–12)	5 (2–11)	0.27
**Age group, yrs**			
0–4	18 (56.3)	245 (45.1)	0.31
5–11	6 (18.8)	169 (31.1)	
12–17	8 (25.0)	129 (23.8)	
**Sex**			
Male	14 (43.8)	313 (57.6)	0.12
Female	18 (56.3)	230 (42.4)	
**Race and ethnicity**			
American Indian or Alaska Native, non-Hispanic	0 (—)	6 (1.2)	0.84
Asian or Pacific Islander, non-Hispanic	1 (3.3)	23 (4.5)	
Black or African American, non-Hispanic	8 (26.7)	149 (29.4)	
Hispanic or Latino	7 (23.3)	139 (27.5)	
White, non-Hispanic	13 (43.3)	183 (36.2)	
Multiracial	1 (3.3)	6 (1.2)	
**Influenza vaccination status** ^§^			
Vaccinated	4 (17.4)	190 (42.4)	0.02
Not vaccinated	19 (82.6)	258 (57.6)	
Ineligible	5 (0.0)	46	
**Influenza test type** ^¶^			
Rapid antigen	0 (—)	8 (1.5)	0.77
RT-PCR	32 (100.0)	517 (95.2)	
Rapid PCR	1 (3.1)	19 (3.5)	
**Influenza type**			
A	30 (93.8)	530 (97.6)	<0.01
B	0 (—)	11 (2.0)	
A and B	2 (6.3)	2 (0.4)	
**RSV test result**			
Positive	3 (10.7)	6 (1.3)	<0.01
Negative	25 (89.3)	460 (98.7)	
**Reason for admission**			
Influenza-related illness	26 (83.9)	436 (81.2)	0.68
Obstetrics or labor and delivery admission	0 (—)	4 (0.7)	
Inpatient surgery procedures	0 (—)	3 (0.6)	
Psychiatric admission needing acute medical care	1 (3.2)	4 (0.7)	
Trauma	0 (—)	10 (1.9)	
Other	4 (12.9)	80 (14.9)	
**Any underlying medical condition**	18 (56.3)	317 (58.4)	0.81
Chronic lung disorder	1 (3.2)	35 (6.8)	0.49
Chronic metabolic disorder	4 (12.9)	30 (5.9)	0.10
Blood disorder	0 (—)	35 (6.8)	0.14
Cardiovascular disorder	2 (6.5)	25 (4.9)	0.67
Neurologic disorder	4 (12.9)	88 (17.2)	0.58
Immunocompromised condition	0 (—)	38 (7.4)	0.12
Renal disease	0 (—)	9 (1.8)	0.46
Gastrointestinal or liver disease	0 (—)	5 (1.0)	0.59
Rheumatologic, autoimmune, or inflammatory conditions	0 (—)	2 (0.4)	0.73
Hypertension	0 (—)	7 (1.4)	0.52
Obesity	1 (5.0)	57 (13.8)	0.18
Pregnant**	0 (—)	0 (—)	—
**Received influenza antiviral treatment** ^††^	17 (53.1)	326 (60.0)	0.44
**Admitted to ICU**	10 (31.3)	117 (21.5)	0.20
**Invasive mechanical ventilation**	4 (12.5)	23 (4.2)	0.03
**BiPAP or CPAP use**	5 (15.6)	35 (6.4)	0.05
**High flow nasal cannula**	5 (15.6)	57 (10.5)	0.36
**Vasopressor use**	3 (9.4)	20 (3.7)	0.11
**Renal replacement therapy or dialysis**	0 (—)	2 (0.4)	0.73
**In-hospital deaths**	0 (—)	0 (—)	—

**Abbreviations:** BiPAP = bilevel positive airway pressure; CPAP = continuous positive airway pressure; ICU = intensive care unit; PCR = polymerase chain reaction; RSV = respiratory syncytial virus; RT-PCR = reverse transcription–polymerase chain reaction.

* Data on race and ethnicity were unknown for two (6.3%) patients with influenza and SARS-CoV-2 coinfection and 37 (6.8%) patients with only influenza; data on current season influenza vaccine were unknown for four (12.5%) patients with influenza and SARS-CoV-2 coinfection and 49 (9.0%) with only influenza; data on RSV test results were unknown or missing for four (12.5%) patients with influenza and SARS-CoV-2 coinfection and 77 (14.2%) patients with only influenza; and data on reason for admission were unknown or missing for one (3.1%) patient with influenza and SARS-CoV-2 coinfection and six (1.1%) patients with only influenza.

^†^ Medians were compared using a Wilcoxon rank sum test. Proportions were compared using Pearson's chi-square tests.

^§^ Vaccinated is defined as immunization in a person aged ≥6 months who received ≥1 dose of the current season’s vaccine ≥14 days before positive influenza test date; a person is considered ineligible if aged <6 months.

^¶^ Proportions for test types are not mutually exclusive.

** Only among adolescents aged 15–17 years.

^††^ Influenza antiviral treatments included oseltamivir, peramivir, or zanamivir.

**Deaths**. Forty-four influenza-associated pediatric deaths were reported to the Influenza-Associated Pediatric Mortality Surveillance System during the 2021–22 influenza season, including seven (16%) decedents who had SARS-CoV-2 coinfection (Figure). Among influenza vaccine–eligible children who died and for whom data were available, zero of six with coinfections and five (16%) of 31 with influenza had been vaccinated against influenza (p = 0.57) (Table 2). The most common complications among decedents with coinfections were pneumonia, acute respiratory distress syndrome, and bronchiolitis. Among decedents with influenza, the most common complications were pneumonia, seizures, and acute respiratory distress syndrome. Cardiomyopathy or myocarditis occurred in five (16%) of 32 decedents with influenza and none with coinfection (p = 0.57). One or more underlying medical conditions were reported for four of five children with coinfections who died and 21 (58%) of 36 with influenza (p = 0.63). Influenza antiviral therapy was administered to one child with a coinfection who died and 17 (46%) decedents with influenza (p = 0.21).

**TABLE 2:** Characteristics of children and adolescents aged <18 years who died with influenza and influenza and SARS-CoV-2 coinfections (N = 44) — Influenza-Associated Pediatric Mortality Surveillance System, United States, October 3, 2021–October 1, 2022*

Characteristic	No. of patients (%) with influenza and SARS-CoV-2 coinfection (n = 7)	No. of patients (%) with only influenza (n = 37)	p-value^†^
**Age, yrs, median (IQR) **	6 (2–13)	4 (1–8)	0.34
**Age group, yrs**			
0–4	2 (28.6)	21 (56.8)	0.41
5–11	3 (42.9)	9 (24.3)	
12–17	2 (28.6)	7 (18.9)	
**Sex**			
Male	4 (51.7)	15 (40.5)	0.44
Female	3 (42.9)	22 (59.5)	
**Race and ethnicity**			
American Indian or Alaska Native, non-Hispanic	0 (—)	1 (2.8)	0.66
Asian or Pacific Islander, non-Hispanic	0 (—)	1 (2.8)	
Black or African American, non-Hispanic	0 (—)	6 (16.7)	
Hispanic or Latino	2 (33.3)	8 (22.2)	
White, non-Hispanic	4 (66.7)	18 (50.0)	
Multiracial	0 (—)	2 (5.6)	
**Influenza vaccination status** ^§^			
Fully vaccinated	0 (—)	5 (16.1)	0.57
Not fully vaccinated	6 (100.0)	26 (83.9)	
Ineligible	0 (—)	2	
**Influenza test type** ^¶^			
Rapid antigen	2 (28.6)	9 (24.3)	0.66
RT-PCR	5 (71.4)	31 (83.8)	
**Influenza type**			
A	6 (85.7)	36 (97.3)	0.30
B	1 (14.3)	1 (2.7)	
A and B	0 (—)	0 (—)	—
Other viral coinfection**	1 (14.3)	1 (2.7)	0.33
**ACIP-defined high-risk condition** ^††^			
Yes	4 (80.0)	21 (58.3)	0.63
No	1 (20.0)	15 (41.7)	
**Type of ACIP-defined high-risk condition** ^§§^			
Neurologic disorders	2 (40.0)	12 (33.3)	—
Cardiac and congenital heart diseases	0 (—)	4 (11.1)	—
Pulmonary diseases (including asthma and cystic fibrosis)	3 (60.0)	5 (13.9)	—
Endocrine diseases (including diabetes mellitus)	1 (20.0)	2 (5.6)	—
Premature at birth	0 (—)	2 (5.6)	—
Immunosuppressive conditions	0 (—)	1 (2.8)	—
Renal diseases	1 (20.0)	0 (—)	—
Genetic disorders	2 (40.0)	6 (16.7)	—
Mitochondrial disorders	0 (—)	1 (2.8)	—
Obesity	0 (—)	2 (5.6)	—
**Received influenza antiviral treatment** ^¶¶^	1 (14.3)	17 (45.9)	0.21
**Hospitalized**			
Yes	4 (57.1)	21 (56.8)	1.00
No	3 (42.9)	16 (43.2)	
**Invasive mechanical ventilation**			
Yes	2 (50.0)	20 (95.2)	0.06
No	2 (50.0)	1 (4.8)	
**Any complication**			
Yes	7 (100.0)	27 (84.4)	0.56
No	0 (—)	5 (15.6)	
**Complications**			
Pneumonia	3 (42.9)	9 (28.1)	0.41
Acute respiratory distress syndrome	2 (28.6)	6 (18.8)	0.61
Croup	0 (—)	2 (6.3)	1.00
Seizures	0 (—)	7 (21.9)	0.32
Bronchiolitis	2 (28.6)	4 (12.5)	0.28
Encephalopathy or encephalitis	0 (—)	4 (12.5)	1.00
Cardiomyopathy or myocarditis	0 (—)	5 (15.6)	0.57
Hemorrhagic pneumonia or pneumonitis	0 (—)	1 (3.1)	1.00
Reye syndrome	0 (—)	0 (—)	—
Shock	1 (14.3)	5 (15.6)	1.00
Sepsis	0 (—)	5 (15.6)	0.56
Other complications	3 (42.9)	13 (40.6)	1.00
**Days from illness onset to death**			
≤1	1 (20.0)	3 (9.7)	0.78
2–7	3 (60.0)	20 (64.5)	
>7	1 (20.0)	8 (25.8)	
**Death location**			
ED	1 (14.3)	7 (18.9)	1.00
ICU	4 (57.1)	19 (51.3)	
Inpatient ward	0 (—)	2 (5.4)	
Outside of hospital	2 (28.6)	9 (24.3)	

**Abbreviations:** ACIP = Advisory Committee on Immunization Practices; ED = emergency department; ICU = intensive care unit; RT-PCR = reverse transcription–polymerase chain reaction.

* Data on race and ethnicity were unknown for one patient with influenza and SARS-CoV-2 coinfection and one (2.7%) patient with only influenza; data on current season influenza vaccine were unknown for one patient with influenza and SARS-CoV-2 coinfection and four (10.8%) patients with only influenza; data on ACIP-defined high-risk conditions were unknown for two patients with influenza and SARS-CoV-2 coinfection and one (2.7%) patient with only influenza; data on invasive mechanical ventilation were unknown for one patient with influenza and SARS-CoV-2 coinfection and one (2.7%) patient with only influenza; data on any complications was unknown for five (13.5%) patients with only influenza; data on days from illness onset to death were unknown for two patients with influenza and SARS-CoV-2 coinfection and six (16.2%) patients with only influenza.

^†^ Medians were compared using a Wilcoxon rank sum test. Proportions were compared using Fisher’s exact tests.

^§^ Fully vaccinated is defined as immunization in a person aged ≥9 years who received ≥1 dose of current season’s vaccine ≥14 days from illness onset; or for a person aged 6 months–8 years who 1) received ≥1 dose of current season’s vaccine ≥14 days from illness onset, and 2) received ≥2 total doses in their lifetime (2 doses of current season’s vaccine, both ≥14 days from illness onset, or 1 dose of current season’s vaccine ≥14 days from illness onset plus 1 dose from a previous season). Not fully vaccinated: a person aged ≥6 months who did not receive any doses of the current season’s vaccine; or a person aged ≥6 months who received ≥1 dose of the current season’s vaccine, but the dose or final dose, if multiple doses, was ≤14 days from illness onset; or for a person aged 6 months–8 years who received only 1 dose of current season’s vaccine ≥14 days from illness onset, but received no other doses from a previous season. A person was considered ineligible if aged <6 months.

^¶^ Proportions for test types are not mutually exclusive.

** One child with a SARS-CoV-2 infection also received a positive test result for adenovirus, rhinovirus/enterovirus, and respiratory syncytial virus. One child with only influenza also received a positive adenovirus test result.

^††^ Children who have chronic pulmonary (including asthma and cystic fibrosis), cardiovascular (excluding isolated hypertension), renal, hepatic, neurologic, hematologic, or metabolic disorders (including diabetes mellitus); are immunocompromised for any reason; are receiving aspirin- or salicylate-containing medications and might be at risk for Reye syndrome after influenza virus infection; or who have extreme obesity.

^§§^ p-values not calculated because of small numbers.

^¶¶^ Influenza antiviral treatments included oseltamivir or peramivir.

## Discussion

The 2020–21 influenza season, which occurred during the COVID-19 pandemic, was characterized by historically low influenza circulation (*6*). However, an unusually late increase in influenza activity occurred in April 2022 during the 2021–22 season (*7*). In this analysis of 2021–22 influenza data from three CDC surveillance systems, among all pediatric patients who received testing for both influenza and SARS-CoV-2 viruses and who were hospitalized or died with influenza, most had underlying medical conditions and were not fully vaccinated against seasonal influenza. Influenza and SARS-CoV-2 coinfections were infrequent (representing 6% of hospitalizations and 16% of deaths within these populations), likely in part because of lower-than-usual influenza virus circulation. However, these data identified increased use of invasive and noninvasive mechanical ventilation among coinfected patients, indicating potentially more severe disease among children and adolescents with influenza and SARS-CoV-2 coinfection. These findings also highlight the underuse of influenza antivirals and seasonal influenza vaccines, particularly among persons aged <18 years with influenza virus and SARS-CoV-2 coinfections who died.

These findings represent a small number of cases of influenza and SARS-CoV-2 coinfection, thereby limiting the ability to draw firm conclusions. The high degree of cocirculation of multiple respiratory viruses during the current season (*1**,**2*), and the higher-than-usual early-season influenza activity, underscore the importance of increasing awareness among parents and providers that influenza and SARS-CoV-2 coinfections occur in pediatric patients and that coinfection can potentially cause more severe illness. For pediatric patients with acute respiratory illness symptoms with suspected severe illness, testing for both influenza and SARS-CoV-2, and other respiratory viruses is critical to facilitate early detection of coinfections and help guide clinical treatment and management (*8*).

The findings in this report are subject to at least six limitations. First, viral testing was performed at the clinician’s discretion or according to hospital policy and might have been influenced by factors including clinical presentation, severity of illness, and previous testing. Both influenza-only and SARS-CoV-2 coinfection cases were not detected if testing for influenza virus and SARS-CoV-2 was not performed for patients with acute respiratory illness. However, coinfected patients might be overrepresented in these results among patients with more severe disease (e.g., on respiratory support) if they were more likely to have been tested for both influenza virus and SARS-CoV-2. Second, information on COVID-19 vaccination and SARS-CoV-2 antiviral treatment was not included because this information could not be systematically ascertained for patients across all data sources. Third, whereas the Influenza-Associated Pediatric Mortality Surveillance System reflects data across all U.S. states and territories, FluSurv-NET and COVID-NET catchment areas include approximately 9%–10% of the U.S. population, limiting the generalizability of results. Fourth, circulation of influenza A and B viruses was lower during 2021–22 than during pre–COVID-19 seasons, thus reducing the number of patients included in the analysis and limiting the ability to examine the clinical effects of COVID-19 on the clinical course of influenza. Ongoing surveillance can help to assess the clinical progression and associated severity of pediatric influenza and SARS-CoV-2 coinfections. Fifth, because of the variability in testing practices found in passive surveillance systems such as the Influenza-Associated Pediatric Mortality Surveillance System (e.g., influenza testing not being performed or being performed late in the course of the illness when influenza could not be detected), pediatric deaths were likely underreported. Finally, SARS-CoV-2–only infections were not reported because these data were not available in the Influenza-Associated Pediatric Mortality Surveillance System.

To prevent and mitigate the incidence of severe respiratory virus–associated illness during periods of influenza virus and SARS-CoV-2 cocirculation, the public and parents should be aware of the risk for pediatric coinfection and adopt prevention strategies, including considering wearing well-fitted, high-quality masks when respiratory virus circulation is high and annual influenza vaccination and up-to-date COVID-19 vaccination (*9**,**10*). To identify coinfections with influenza virus and SARS-CoV-2, clinicians should follow recommended testing algorithms for patients with acute respiratory illness symptoms in outpatient, emergency department, and hospital settings. Clinical guidance on early initiation of antiviral treatment for influenza and SARS-CoV-2 should be followed for pediatric patients with suspected or confirmed influenza or SARS-CoV-2 infections (or both), who are hospitalized, have severe or progressive disease, or are at increased risk for complications (*9*,*10*).

**Summary**

**What is already known about this topic?**

Influenza and SARS-CoV-2 viruses individually contribute to pediatric morbidity. The prevalence and severity of coinfection with influenza and SARS-CoV-2 are less well understood.

**What is added by this report?**

During the 2021–22 influenza season, 6% of hospitalized pediatric influenza patients had SARS-CoV-2 coinfection; a higher percentage of patients with coinfection required invasive or noninvasive respiratory support compared with those with influenza only. Among influenza-associated pediatric deaths, 16% had SARS-CoV-2 coinfection; only one coinfected decedent received influenza antivirals, and none had been fully vaccinated against influenza.

**What are the implications for public health practice?**

The public should adopt prevention strategies, including influenza and COVID-19 vaccination, and consider mask use during high respiratory virus circulation.

## Footnotes

* These authors contributed equally to this report.

† These senior authors contributed equally to this report.

§ Data from FluSurv-NET beyond April 30, 2022, did not include variables required to determine influenza and SARS-CoV-2 coinfection status.

¶
https://www.cdc.gov/flu/weekly/overview.htm#PediatricMortality

** The 2021–22 influenza season was defined as *MMWR *week 40 of 2021 through *MMWR* week 39 of 2022 (October 3, 2021–October 1, 2022). https://ndc.services.cdc.gov/wp-content/uploads/MMWR_Week_overview.pdf

†† Receipt of COVID-19 treatment was not collected on patients in this report.

§§ FluSurv-NET data from the 2021–22 influenza season included county data from 13 U.S. states: California, Colorado, Connecticut, Georgia, Maryland, Michigan, Minnesota, New Mexico, New York, Ohio, Oregon, Tennessee, and Utah. Data from Iowa were excluded from FluSurv-NET during this season because of lack of capture of all required variables. COVID-NET data included all contributing FluSurv-NET counties as well as statewide surveillance data from Maryland.

¶¶ Influenza testing for FluSurv-NET includes rapid antigen, molecular assay, rapid molecular assay, immunofluorescence assay, viral culture, or serology. COVID-19 testing includes rapid antigen, molecular assay, or serology.

*** Trained staff members reviewed laboratory records, medical charts, and reportable conditions databases to identify eligible patients. Data on demographics, clinical characteristics, in-hospital interventions, and clinical outcomes were abstracted using standardized case report forms.

††† Ascertainment of vaccination status was performed using hospital records, state immunization registries, primary care provider surveys, and patient or proxy interview.

§§§ Influenza testing for influenza-associated pediatric mortality includes commercial rapid diagnostic tests, viral culture, fluorescent antibody, enzyme immunoassay, reverse transcription–polymerase chain reaction, and immunohistochemistry.

¶¶¶ 45 C.F.R. part 46.102(l)(2); 21 C.F.R. part 56; 42 U.S.C. Sect. 241(d); 5 U.S.C. Sect. 552a; 44 U.S.C. Sect. 3501 et seq.
